# Molecular basis of trypsin's autolysis resistance acetylation for sustained enzymatic activity

**DOI:** 10.1016/j.fochx.2026.103619

**Published:** 2026-02-06

**Authors:** Xiaozhan Qu, Tengfei Liu, Yalong Xu, Chen Wang, Xueao Zheng, Yixiao Zhang, Peijian Cao, Qiansi Chen

**Affiliations:** a Zhengzhou Tobacco Research Institute of CNTC, Zhengzhou 450001, China; b College of Food Science and Engineering, Shandong Agricultural University, Tai'an 271018, Shandong, China; c Beijing Life Science Academy (BLSA), Beijing 102209, China

**Keywords:** Trypsin, Autolysis, Acetylation, Molecular dynamics simulations, Enzyme stability, Protein engineering

## Abstract

Acetylation serves as an effective strategy to enhance trypsin's resistance to autolysis, yet the underlying molecular mechanism remains unclear. Integrating molecular dynamics (MD) simulations and biochemical assays, we show that acetylation induces global stabilization (RMSD decreased by 0.03 nm) coupled with structural expansion (Rg increased by 0.01 nm) and a significant (*p* < 0.05) increase in local flexibility. These perturbations propagate allosterically to the active site, resulting in its precise structural distortion. Experimentally, acetylated trypsin exhibited markedly improved stability, retaining 80.78% of its activity after six hours of autolysis versus only 54.2% for the native enzyme, despite an initial activity reduction of 23.2%. The molecular basis for this trade-off is an allosterically rewired state that enhances structural integrity while slightly misaligning catalytic residues and promoting a low-efficiency substrate binding mode. Collectively, our work provides atomic-level insights useful for rationally designing trypsin variants with optimized performance in food enzyme engineering.

## Highlights

• Lysine acetylation enhances trypsin's autolytic resistance at the expense of a modest reduction in activity.
• Acetylation induces global structural stabilization coupled with local flexibility and active site distortion.
• Allosteric propagation of perturbations disrupts the catalytic triad geometry.
• A catalytically suboptimal substrate-binding mode with altered hydrogen bonding is formed in acetylated trypsin.

## 1 Introduction

**Fig. 1:**
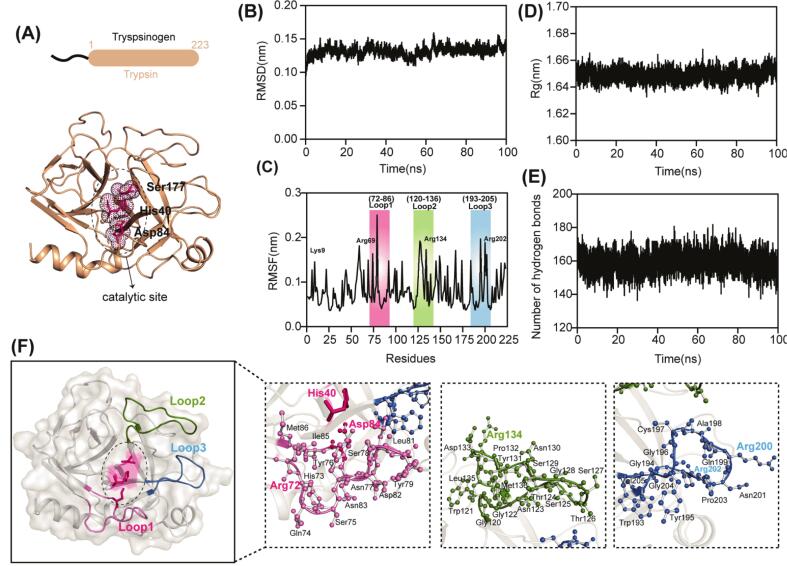
**Molecular dynamics analysis of the trypsin autoproteolytic activity. (A)** Structure of trypsin (obtained from AlphaFold3) with catalytic residues (His40, Asp84 and Ser177) highlighted in hotpink sticks. **(B)** RMSD of trypsin over a 100 ns simulation, reflecting structural stability. **(C)** RMSF highlighting regions of local flexibility; key loop regions (loop1: 72–86, loop2: 120–136, loop3: 193–205) and autolysis-prone residues (Lys9, Arg69, Arg134, Arg202) are indicated. **(D)** Rg of trypsin during the 100 ns simulation, reflecting changes in structural compactness. **(E**) Number of hydrogen bonds of trypsin during 100 ns simulation. **(F)** Structural overview highlighting dynamic regions and key residue interactions involved in autoproteolysis.

Trypsin, a serine protease essential for digestive and cellular regulatory functions, exhibits high substrate specificity and catalytic efficiency. It is initially synthesized in the pancreas as an inactive proenzyme, known as trypsinogen, which is proteolytically cleaved to generate active trypsin (Ma et al., 2020; Najar et al., 2021). The mature enzyme consists of 223 amino acid residues folded into two six-stranded β-barrels and features a conserved catalytic triad (aspartic acid, histidine, serine) (Fig.1A)(Aggarwal & Ikram, 2022). Critically, trypsin possesses strict substrate specificity, exclusively facilitating the hydrolysis of only lysine (Lys) and arginine (Arg) bonds through an ionic binding site within its active site (Li et al., 2022). This specificity underpins its indispensable roles in diverse contexts, including physiological processes, apoptosis, signal transduction, homeostasis, and immune regulation, as well as its broad applications in the food industry for protein hydrolysis in products including infant formula, nutraceuticals, and food additives(Ge et al., 2021; Pang et al., 2021; Peng et al., 2019; Qi et al., 2023; Zhang et al., 2020).

However, A major limitation to trypsin's utility is its susceptibility to autolysis—a self-degradation process in which trypsin cleaves its own peptide bonds, leading to irreversible inactivation (Hedstrom, 2002). This autolytic behavior complicates industrial applications such as biocatalysis and protein processing, and poses significant challenges in therapeutic contexts where extended enzyme stability is essential(Ensign et al., 2012; Gupta et al., 2002). Various strategies have been developed to reduce autolysis and enhance trypsin stability. These include chemical modifications like PEGylation (Zarafshani et al., 2010) or polymer conjugation (Chanphai et al., 2017; Chanphai & Tajmir-Riahi, 2016; Chiu et al., 2010), which can sterically shield cleavage sites but may affect substrate binding and turnover. Immobilization onto solid supports can reduce autolytic access but often at the cost of reduced activity and mass transfer limitations (Anirudhan & Rejeena, 2012; Caro et al., 2010). Protein engineering approaches, including site-directed mutagenesis of identified autolysis hotspots, have yielded more stable variants, though these alterations may affect catalytic properties or specificity(Zhang et al., 2016). In this context, post-translational modifications (PTMs) represent a promising alternative, as they offer a natural and often reversible mechanism for regulating protein structure, activity, and stability, potentially enabling finer control than exogenous interventions (Zhong et al., 2023).

Among PTMs, lysine acetylation has emerged as a potent modulator of protein conformation and proteolytic resistance across diverse systems (Shaw et al., 2008). Although prior studies have identified key autolytic sites in trypsin (Enoksson et al., 2007; Yamamoto et al., 2017), the mechanistic relationship between autolysis and regulatory PTMs such as acetylation remains poorly understood. It has been proposed that acetylation may reduce autolysis by sterically blocking cleavage sites or by stabilizing tertiary structure (Zhao et al., 2015). However, structural insights have largely been derived from static X-ray crystallographic models, which cannot capture the dynamic processes underlying autolysis or the transient effects of PTMs. Thus, there is a lack of direct experimental evidence mechanistically linking lysine acetylation to structural dynamics, autolysis kinetics, and functional stability in trypsin that hinders the exploitation of this PTM for industrial or therapeutic applications.

Molecular dynamics (MD) simulation offers a powerful computational framework for probing the dynamic behavior of biomolecules at atomic resolution and across biologically relevant timescales in food engineering(Wei et al., 2024; Xue et al., 2022; Zhang et al., 2024). It is particularly well-suited to capture transient conformational changes, such as those induced by lysine acetylation that occur on the nanosecond-to-microsecond scale. Recent advances in MD methodologies have enabled detailed investigations into enzymatic mechanisms and the rationalization of chemical modification engineering, providing critical atomistic insight into how acetylation can sterically block autolytic sites in trypsin (Chan & Shukla, 2023; Kawade et al., 2020; Lee et al., 2015; Sovová et al., 2020; Weigle et al., 2022; Winogradoff et al., 2015). In this study, we employed MD simulations to systematically investigate the structural dynamics of trypsin autolysis and its modulation by lysine acetylation, both in solution and in ligand-bound states. Our results reveal that acetylation induces global structural stabilization but concurrently leads to active site distortion via long-range allosteric propagation, ultimately resulting in enhanced autolytic resistance at the expense of a modest reduction in activity. These findings not only provide a mechanistic understanding of PTM-mediated enzyme regulation but also establish a rational basis for designing allosterically engineered proteases with tailored stability-activity profiles. The insights offered here could guide novel strategies for optimizing enzymatic performance in biotechnology and medicine, where fine-tuning the balance between stability and activity is essential for functional longevity.

## 2 Materials and methods

### 2.1 Expression and preparation of proteins

The human trypsinogen gene (GenBank accession: 5TP0_A) was codon optimized, synthesized, and inserted into the *Nde*I/*Xho*I-digested pET-16b vector with a 6× His tag and transformed into *E. coli* BL21 (DE3) cells. Transformed cells were grown in LB medium at 37 °C with ampicillin (100 μg/mL). Protein expression was induced by adding 0.3 mM IPTG at OD_600_ ∼ 0.6, followed by 18 to 24 h at 16 °C.

### 2.2 Purification of proteins

Cells were harvested by centrifugation at 5000*g* for 20 min at 4 °C, resuspended in lysis buffer (25 mM Tris-HCl pH 8.0, 500 mM NaCl, 10 mM imidazole, and 1 mM PMSF), and stored at −80 °C until use. After thawing, cells were sonicated at 250 W for 30 min (1 s on, 2 s off) on ice. The lysate was clarified by centrifugation at 50,000*g* for 30 min at 4 °C, and the pellet was resuspended in lysis buffer with 8 M urea (Denaturation buffer) and incubated at 37 °C overnight. The suspension was then centrifuged at 50,000*g* for 30 min at 25 °C. The supernatant was passed through a Ni^2+^-chelating column (GE Healthcare) pre-equilibrated with denaturation buffer. The column was then washed with 25 mM Tris-HCl pH 8.0, 500 mM NaCl, 6 M urea and 25 mM imidazole to remove non-specifically bound proteins, and the target protein was eluted with 25 mM Tris-HCl pH 8.0, 150 mM NaCl, 6 M urea and 300 mM imidazole. The eluted protein was then dialyzed in 1 L of refolding buffer (20 mM Tris, 2 M urea, 1 mM cystine, 3 mM cysteine, pH 7.5) at 4 °C overnight.

### 2.3 Activation and purification of trypsin

The purified trypsinogen was concentrated and exchanged into activation buffer (25 mM Tris-HCl, pH 8.0) using an Amicon Ultra 15 mL concentrator (10 kDa Centrifugal Filter Unit, Merck Millipore). After 1 h of activation at room temperature with 0.02% (*V*/V) Enterokinase, the protein mixture was then subjected to another round of Ni^2+^-chelating column purification to remove the amino-terminal propeptide and protease. The flow-through was collected, concentrated to 5 mL and applied to a HiLoad 16/600 Superdex 200 size exclusion column (GE Healthcare) with buffer containing 50 mM Tris and 10 mM CaCl_2_ at pH 8.0.

### 2.4 Expression, purification, and biochemical characterization of recombinant trypsin

To experimentally validate the impact of acetylation on trypsin, we conducted comprehensive biochemistry experiments to assess its stability and catalytic activity. Recombinant trypsin was expressed in *E. coli* BL21 and SDS-PAGE showed that the expressed product existed mainly in the inclusion bodies (IB) (Fig.S1A). We developed a systematic methodological process for purifying trypsin IB (Fig.S1B). After sonication and centrifugation, the IB was solubilized in denaturation buffer, purified by Ni^2+^-NTA affinity column and the elution peak was refolded through dialysis (Fig.S1C). After activation, the trypsinogen mixture was subjected to a second round of Ni^2+^-chelating column purification to remove the amino-terminal tag and Enterokinase (Fig. S1D). The flow-through was collected, concentrated, and applied to a HiLoad 16/600 Superdex 200 size-exclusion column (Fig.S1E). The SDS-PAGE analysis of the purified trypsinogen and activated trypsin showed, as predicted, that the trypsin band was slightly smaller than that of trypsinogen because the propeptide cleavage (Fig.3A). The results indicated that trypsin exhibited detectable degradation, with a faint band appearing below the main trypsin band, suggesting partial proteolysis.

### 2.5 Acetylation of trypsin

The acetylation was performed according to the method described previously (Zhao et al., 2015): the sample was buffer exchanged into 50 mM sodium acetate-acetic acid buffer at pH 5.5 containing 20% glycerol. Acetic anhydride (analytical grade) was diluted to 800 mM with dioxane (Sigma-Aldrich) before addition to the r-trypsin sample in a 5 mL/L reaction volume with stirring to start the acetylation reaction. The pH was stabilized to 6.5 by 0.5 M sodium hydroxide and maintained for 30 min. The acetylated trypsin was henceforth referred to as Ac-trypsin. Then the sample was concentrated and applied to a HiLoad 16/600 Superdex 200 size exclusion column (GE Healthcare) with buffer containing 50 mM Tris, 10 mM CaCl_2_, pH 8.0.

### 2.6 Determination of SDS-PAGE

To evaluate their stability, trypsin and Ac-trypsin were diluted to 10 μM with 50 mM ammonium bicarbonate and incubated at 37 °C for 30 min. Samples were taken every 10 min and analyzed by electrophoresis on a 15% SDS-PAGE gel under denaturing conditions.

### 2.7 Determination of enzyme activity

The activity of Ac – trypsin was measured using an N - Benzoyl - L - arginine ethyl ester (BAEE) - based assay. BAEE is a canonical, chromogenic substrate specifically designed for trypsin and related serine proteases. The choice of this assay over alternative methods (e.g., casein hydrolysis) was based on its superior sensitivity, specificity, and dynamic range for precise kinetic comparison. Briefly, the activity was measured by monitoring the initial increase in absorbance at 253 nm (ΔA₂₅₃/min) in a reaction system containing 50 mM Tris-HCl (pH 8.0) and 10 mM CaCl₂. One unit of enzyme activity (U) was defined as the amount of enzyme required to produce an absorbance increase of 0.001 per minute under the assay conditions. As a control, a commercial bovine trypsin (Macklin, T819002) was measured.

### 2.8 Circular dichroism spectroscopy

Circular dichroism (CD) experiments were conducted using Chirascan V100 spectromet (Applied Photophysics) at 25 °C. Circular dichroism spectra were recorded in the wavelengths between 260 and 190 nm, with a 2.0 mm path length cell, 1.0 nm bandwidth and a response time of 2 s, averaging three accumulations. The trypsin samples were tested at a concentration of 0.3 mg/mL. The spectra are expressed as molar ellipticity.

### 2.9 Mass spectra measurment

To obtain definitive chemical evidence of successful lysine acetylation, the molecular masses of purified native trypsin and Ac-trypsin were determined by matrix-assisted laser desorption/ionization time-of-flight mass spectrometry (MALDI-TOF MS). Samples were prepared by mixing the protein solution (1 mg/mL) with a sinapinic acid matrix solution at a 1:1 (*v*/v) ratio. Then, 1 μL of the mixture was spotted onto a stainless steel target plate and allowed to dry at room temperature. Spectra were acquired in linear positive ion mode. External calibration was carried out using a protein standard mixture. Data processing and deconvolution were performed with the flexAnalysis software (Bruker Daltonics).

### 2.10 Molecular docking

The trypsin sequence was submitted to the AlphaFold3 Server (https://golgi.sandbox.google.com/) to generate a three-dimensional structural model(Abramson et al., 2024). The structure of acetylated trypsin was submitted to the Protenix Server, and the lysine residues within the trypsin sequence were acetylated(https://protenix-server.com/). Molecular docking of BAEE to trypsin and Ac-trypsin was carried out by AutoDock Vina1.2.5 (Eberhardt et al., 2021). The 3D structure of BAEE (PubChem ID:24891777) were obtained from NCBI PubChem. Semi-flexible docking method was used, where trypsin was treated as a rigid body and all rotatable bonds in the BAEE were sampled. Optimal binding sites were searched in a box of 60x60x60 Å^^3^ that covered the entire exterior of the protein. The box had 1.0 Å grid spacing and was centered at the geometric center of the protein. The docking protocol utilized AutoDock Vina's scoring function and search algorithm to generate and evaluate multiple ligand conformations within this search space.The pose exhibiting the lowest binding energy was selected as the optimal docking solution, representing the most favorable predicted interaction between trypsin and Ac-trypsin with BAEE. The detailed contacts between trypsin and Ac-trypsin with BAEE were analyzed using Ligplot(Laskowski & Swindells, 2011),and the interaction map of the trypsin-BAEE complex was generated via PyMOL.

### 2.11 Molecular dynamics (MD) simulations

MD simulations were performed for 100 ns using the GROMACS 2022 package (https://manual.gromacs.org) with Amber ff99SB-ILDN force field and TIP3P explicit water model for trypsin and r-Ac-trypsin with or without substrate. The AMBER ff99SB force field was used to describe the topology and charge of the protein. Subsequently, counter ions were added to neutralize the unbalanced charge of the system. Energy minimization was performed using the steepest descent method. After minimization, the system was then gradually heated to 300 K under NVT conditions with positional restraints on the protein backbone atoms, allowing for equilibration of the solvent and ions. Subsequently, a 100 ps NPT equilibration was performed at 300 K and 1 atm using the V-rescale thermostat and Parrinello-Rahman barostat. All the systems were simulated by MD for 100 ns. All bonds involving hydrogen atoms were constrained using the LINCS algorithm. Trajectories were saved every 2 ps for analysis.

### 2.12 Statistical analysis

All biochemical experiments were performed with at least three independent replicates. Data are presented as mean ± SD. Statistical comparisons between two groups were performed using a two-tailed Student's *t*-test. Differences were considered statistically significant at *p* < 0.05. Statistical analyses and graphical presentations were conducted using GraphPad Prism 8.0 software.

## 3 Results

### 3.1 Trypsin exhibits high flexibility in catalytic regions prone to autolysis

A fundamental question in understanding the trypsin function concerns the structural basis of its autolysis behavior. In this study, the catalytic triad residues of trypsin were identified as Ser177, His40, and Asp84 (Fig.S2A). Autolysis involves the proteolytic cleavage of specific arginyl and lysyl peptide bonds within trypsin, leading to a progressive loss of enzymatic activity. In human trypsin, the acetylated sites include 5 lysine and 6 arginine residues (Fig.S2B). To investigate the conformational mechanism underlying trypsin autolysis, we performed 100 ns MD simulations to characterize the structural properties of native trypsin (Yeggoni et al., 2017). The root-mean-square deviation (RMSD) is a key metric for evaluating structural stability and dynamic behavior during simulations (da Fonseca et al., 2024). After an initial 10 ns equilibration period, the trypsin maintained a stable RMSD value with minimal fluctuations (Fig.1B). The root-mean-square fluctuation (RMSF), which quantifies flexibility by measuring positional variations of individual atoms and local conformational changes (Allen et al., 2004), revealed elevated fluctuations in three loop regions: loop1 (residues 72–86), loop2 (residues 120–136), and loop3 (residues 193–205) (Fig.1C). The most susceptible autolytic sites in trypsin (Lys9, Arg69, Arg134, and Arg202) are located within these flexible loops. Notably, Arg134 and Arg202 reside in active site loops and exhibit remarkable flexibility and dynamics, making them particularly accessible for proteolytic cleavage at the catalytic center (Fig.1F). Analysis of the radius gyration (Rg), which reflects the spatial distribution of atoms relative to the geometric center of the molecule, showed only minor fluctuations through the simulation, indicating overall structure stability (Fig.1D). Additionally, the number of intramolecular hydrogen bonds, critical for maintaining secondary structure, remained largely consistent, confirming the global structural integrity of trypsin (Fig.1E). These findings are consistent with previous MD studies on trypsin, which also reported a stable global fold coupled with high flexibility in catalytically relevant regions (Li et al., 2022; Ma et al., 2020).

### 3.2 Effects of acetylation on the structure and dynamics of trypsin

**Fig. 2:**
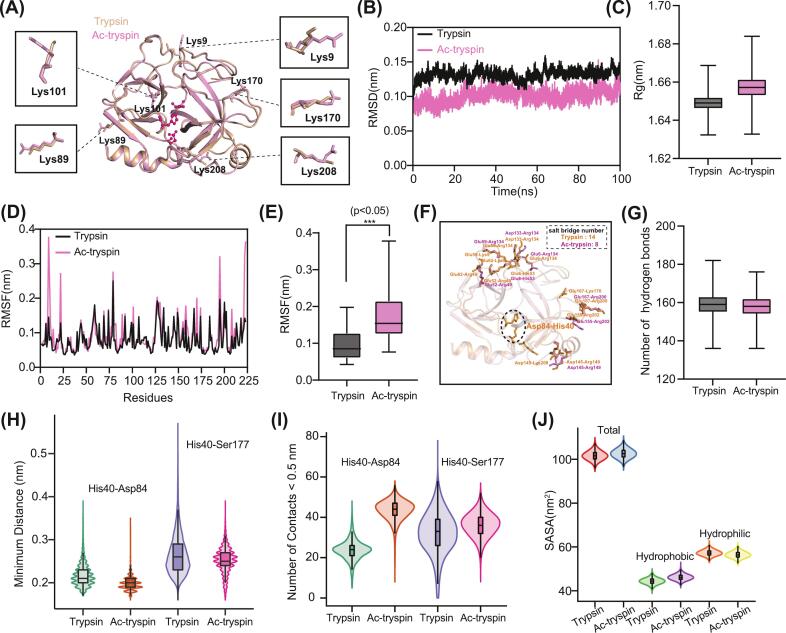
**Molecular dynamics analysis reveals the structural and dynamic perturbations induced by lysine acetylation in trypsin. (A)** Structural alignment of native trypsin (gray) and acetylated trypsin (Ac-trypsin; pink), with acetylated lysine residues (Lys9, Lys89, Lys101, Lys170, and Lys208) highlighted. **(B)** RMSD trajectories demonstrate enhanced global stability in Ac-trypsin (pink) compared to native trypsin (black). **(C)** Rg profiles indicate structural expansion in Ac-trypsin. **(D)** RMSF shows increased flexibility in regions near to acetylated sites. **(E)** Comparative RMSF analysis of residues adjacent to acetylation sites reveals significant increases in local flexibility. Data represent mean ± SD; asterisks indicate significant differences (*p < 0.05). **(F)** Salt bridge analysis shows disruption of key electrostatic interactions in Ac-trypsin, with representative interactions listed and count reduced from 14 to 8. **(G)** Number of intramolecular hydrogen bonds shows modest reduction in Ac-trypsin. **(H)** Minimum distance between catalytic residue pairs His40-Asp84 and His40-Ser177 indicate active site compaction in Ac-trypsin. **(I)** Number of inter-residue contacts within 0.5 nm distance cutoff suggests altered site geometry around catalytic residues pairs. **(J)** SASA expansion in Ac-trypsin correlates with structural relaxation and enhanced surface exposure. (For interpretation of the references to colour in this figure legend, the reader is referred to the web version of this article.)

Acetylation is a common post-translational modification involving the addition of an acetyl group to the ε-amino group of a lysine residues. This reaction converts the positively charged NH₃^+^ into a neutral *N*-acetylated amide (NHCOR), thereby eliminating the positive charge and introducing a relatively small hydrophobic moiety. To investigate the structural consequences of lysine acetylation, we modeled fully acetylated lysine residues within the trypsin sequence and generated acetylated trypsin structures using the Protenix server (https://protenix-server.com/). Structural alignment revealed that acetylation did not induce major conformational changes in the overall protein fold (Fig. 2A). The electrostatic potential on the molecular surface was calculated using the Adaptive Poisson-Boltzmann Solver (APBS) software. Intriguingly, the surface electrostatic potential remained remarkably similar between acetylated and unmodified trypsin (Fig. S2C).

We subsequently characterized the structural and dynamic properties of acetylated trypsin using MD simulations. The RMSD of the backbone atoms was calculated for both modified and unmodified systems over the entire simulation time. After 20 ns, the RMSD values of both systems exhibited slow fluctuations and gradually stabilized between 50 and 100 ns. Although the two systems shared identical initial structures, the acetylated trypsin consistently showed lower RMSD values throughout the simulation compared to the unmodified system (Fig. 2B), suggesting enhanced global stability of the protein core following modification. Paradoxically, this increase in backbone stability was accompanied by a significant increase in the Rg (from 1.649 to 1.657), indicating a more expanded and less compact overall structure (Fig. 2C). This structural expansion was further supported by an 0.72% increase in the total solvent-accessible surface area (SASA) (Fig. 2J), consistent with a more relaxed and open conformation.

The apparent discrepancy between global stability and structural expansion was resolved by analyzing local flexibility and interactions. RMSF analysis revealed a substantial increase in flexibility, particularly in regions containing acetylated residues and surface loops (Fig. 2D, E, and Fig. S3A). We attribute this localized flexibility to the elimination of positive charges upon acetylation, which disrupts pre-existing salt bridges and electrostatic interactions that previously constrained these dynamic regions (Fig. 2F). Concurrently, the total number of hydrogen bonds decreased from 159 to 157 upon acetylation (Fig. 2G). This decline was particularly pronounced for hydrogen bonds involving catalytic site residues, indicating a direct perturbation of the key interactions essential for function (Fig. S3B). Strikingly, inter-residue contacts maps analysis showed no major global structural rearrangement between the two forms (Fig. S3C and 4D). However, analysis of the active site revealed a subtle yet critical distortion. Despite overall protein expansion, the minimum distance between the catalytic residues His40, Asp84, and Ser177 significantly decreased upon acetylation (Fig. 2H). Concurrently, the number of contacts within a 0.5 nm cutoff around the His40-Asp84 and His40-Ser177 pairs increased (Fig. 2I), suggesting compaction and tightening of the active site pocket. Since the catalytic efficiency of trypsin relies on a precise spatial arrangement of these residues, the observed shortening of distances likely disrupts the optimal geometry for proton transfer and substrate stabilization.

This active site distortion represents a clear manifestation of a long-range allosteric effect. The initial perturbation caused by acetylation at lysine residues propagates through the protein scaffold, inducing structural strain that is ultimately relieved by conformational rearrangement within the active site, compromising its functional integrity. We postulate that this acetylation-induced restructuring, characterized by the global expansion, increased loop flexibility, and a locally distorted active site, could have profound implications for the catalytic efficiency and substrate recognition of trypsin.

### 3.3 Acetylation enhances trypsin's resistance to autolysis at the expense of a modest reduction in activity

**Fig. 3:**
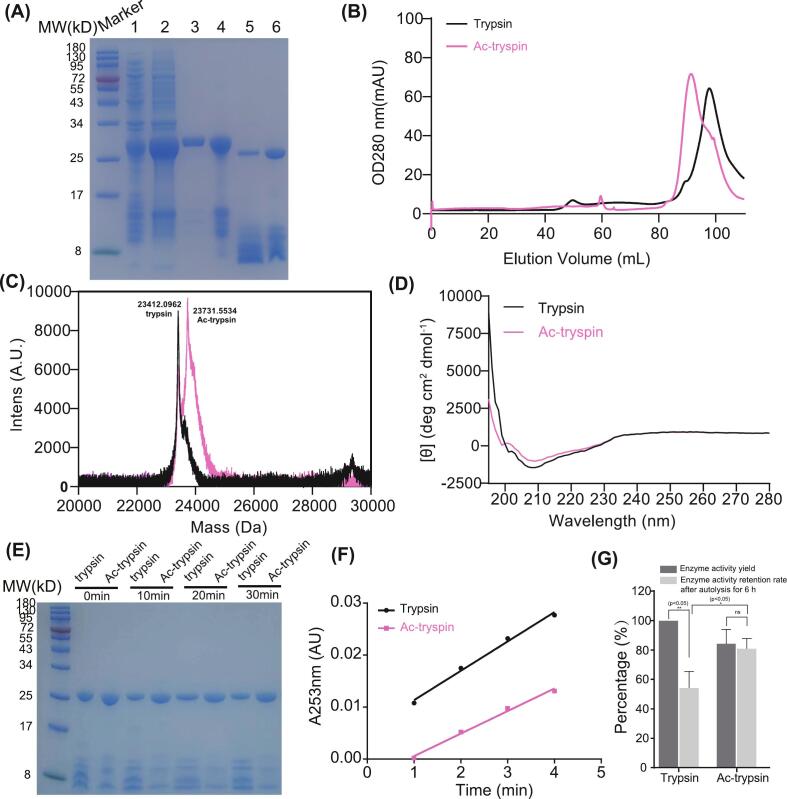
**Experimental validation of acetylation effects on trypsin structure and function. (A)** SDS-PAGE analysis of samples from key purification stages: lane 1, IPTG induction; lane 2, dissolved inclusion bodies; lane 3, the first Ni-NTA affinity purification elution; lane 4, after refolding; lane 5, after activation; lane 6, final purified trypsin. **(B)** Superdex-200 chromatography profiles of Ac-trypsin (pink) and trypsin (black) reveal distinct elution volumes, indicating conformational differences. **(C)** MALDI-TOF mass spectrometry of native trypsin (black) and Ac-trypsin (pink). The measured average molecular masses are labeled. The clear mass shift for Ac-trypsin corresponds to the addition of 5 acetyl groups, providing direct evidence of successful lysine acetylation. **(D)** CD spectra of native trypsin (black) and Ac-trypsin (pink). The near superimposition of the two spectra indicates that lysine acetylation does not induce significant changes in the overall secondary structure of trypsin. **(E)** Autolysis resistance assay of trypsin and Ac-trypsin Incubated at 37 °C for 0, 10, 20, and 30 min. Ac-trypsin exhibits reduced degradation compared to trypsin. **(F)** Enzymatic activity assay using BAEE as substrate. Absorbance was monitored at 253 nm. Specific activities were determined as 423,000 U/mL for trypsin and 324,750 U/mL for Ac-trypsin. **(G)** Comparison of enzymatic activity yield after modification and activity retention rate following 6 h of autolysis. Acetylation significantly improves trypsin's stability while moderately reducing catalytic activity. Data are presented as mean ± SD. The difference (p < 0.05) as indicated asterisks. (For interpretation of the references to colour in this figure legend, the reader is referred to the web version of this article.)

To experimentally validate the computational predictions, native trypsin was purified to homogeneity and subjected to in vitro chemical acetylation using acetic anhydride (Fig.3A). Following acetylation, the sample was purified via gel filtration chromatography. Notably, acetylated trypsin (Ac-trypsin) eluted at 91.3 mL, a lower retention volume compared to unmodified trypsin (97.7 mL) (Fig.3B), suggesting a change in hydrodynamic properties. The mass spectrum of Ac-trypsin (∼23,731 Da) exhibited a distinct shift toward a higher mass relative to the native enzyme (∼23,512 Da), consistent with the addition of 5 acetyl groups (each contributing ∼43 Da) (Fig. 3C). The CD spectra of native and Ac-trypsin were virtually superimposable, exhibiting characteristic minima near 208 nm and 222 nm, which are indicative of a well-folded protein with substantial α-helical and β-sheet content (Fig. 3D). Moreover, the CD spectra remained unchanged upon the addition of the substrate analog BAEE (Fig. S4A), indicating that substrate binding also does not induce major conformational rearrangements in the acetylated enzyme.

We next performed an autolysis assay by incubating 10 μM samples of trypsin or Ac-trypsin in 50 mM Tris-HCl buffer (pH 8.0) containing 10 mM CaCl₂ at 37 °C and analyzing them via SDS-PAGE at 0, 10, 20, and 30 min. The unmodified trypsin showed a significant decrease over time, whereas Ac-trypsin levels remained largely unchanged, indicating markedly improved resistance to autolytic degradation (Fig.3E). These results confirm that lysine acetylation enhances trypsin's stability, making Ac-trypsin a promising candidate for applications requiring prolonged enzymatic activity.

To evaluate the enzymatic activity of the purified Ac-trypsin, we performed a N-Benzoyl-L-arginine ethyl ester (BAEE) assay. Activity was determined by monitoring the rate of increase in absorbance at 253 nm, which signifies the hydrolysis of BAEE to benzoyl-arginine (BA). The activity of native trypsin was measured as 423,000 U/mL, compared to 324,750 U/mL for Ac-trypsin (Fig.3F), indicating a moderate activity reduction of 23.2%. Intriguingly, after 6 h of autolysis, the activity retention of the trypsin decreased markedly to 54.2% of its original level. In contrast, Ac-trypsin demonstrated significantly enhanced stability, retaining 80.78% of its post-modification activity under the same conditions (Fig.3G). This indicates a 26.58% higher retention rate for the acetylated enzyme, clearly demonstrating the substantial functional improvement brought about by acetylation. These findings suggest that Ac-trypsin shows considerable potential for industrial applications where sustained enzyme function is crucial.

To confirm that the observed activity reduction is an inherent effect of the acetylation chemistry and not specific to our recombinant protein, commercial trypsin was also acetylated. The commercial enzyme had a lower baseline activity (279,750 U/mL), which further decreased to 267,000 U/mL after acetylation, exhibiting a proportional loss consistent with that of the recombinant variant (Fig. S4B, C). This result reinforces that the attenuating effect on catalysis is generalizable across enzyme sources.

### 3.4 Molecular docking analysis of binding between trypsin and Ac-trypsin with substrate

**Fig. 4:**
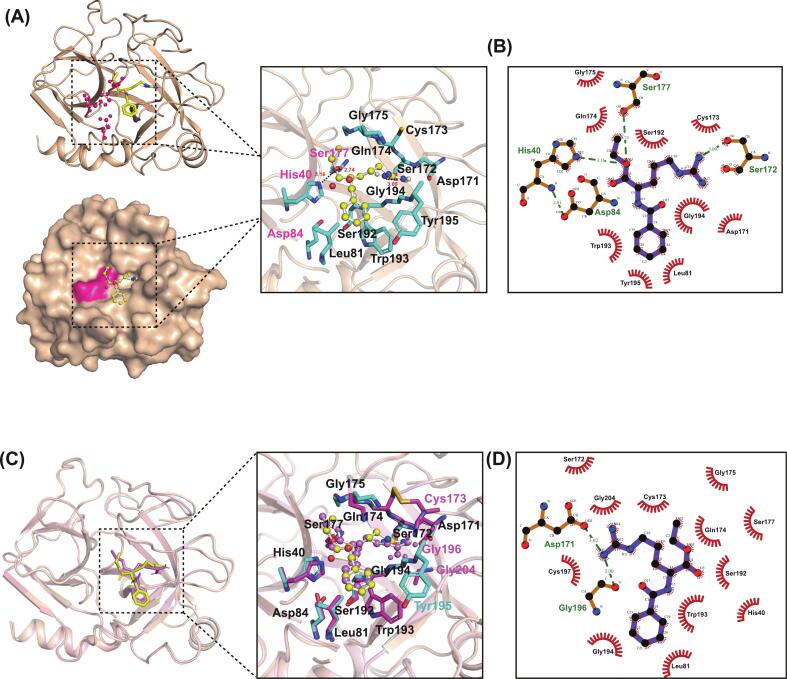
**Molecular docking analysis of BAEE binding to native trypsin and Ac-trypsin. (A)** Structural model of the trypsin-BAEE complex. Trypsin is shown in wheat cartoon (top) and surface (bottom) representations; BAEE is depicted in yellow ball-and-stick. The right panel shows a detailed view of BAEE bound within the catalytic pocket, with key interacting residues in cyan ball-and-stick. Hydrogen bonds are depicted with black dashed lines. **(B)** Two-dimensional interaction diagram (Ligplot) of BAEE with residues in trypsin. **(C)** Structural superposition of the trypsin-BAEE (yellow) and Ac-trypsin-BAEE (magenta) complexes. The right panel compares the binding modes of BAEE in the catalytic pockets, with key residues colored cyan (trypsin) and magenta (Ac-trypsin). **(D)** Ligplot depicting the interaction diagram of BAEE with residues in Ac-trypsin. (For interpretation of the references to colour in this figure legend, the reader is referred to the web version of this article.)

To elucidate the molecular mechanism by which acetylation reduces enzymatic activity, we investigated the structural determinants of BAEE (a synthetic chromogenic substrate specifically hydrolyzed by trypsin, was used as the ligand for this study.) binding in native trypsin and Ac-trypsin using molecular docking. The calculated binding energy (ΔG) for the trypsin-BAEE complex was −6.981 kcal/mol), lower than that of the Ac-trypsin-BAEE complex (−6.576 kcal/mol), suggesting stronger binding affinity in the unmodified enzyme. Structural analysis revealed that the alkyl hydrophobic chain of BAEE inserts into the canonical S1 pocket near the active site (Fig.4A), while its polar group approaches His40 and Ser177, both critical for catalysis. Detailed interactions analysis using LigPlot showed that BAEE is surrounded by residues His40, Leu81, Asp84, Asp171, Ser172, Cys173, Gln174, Gly175, Ser177, Ser192, Trp193, Gly194, and Tyr195 in native trypsin (Fig.4B). Critically, hydrogen bonds were formed between BAEE with His40 and Ser177 of trypsin, which are key components of the catalytic triad, facilitating optimal substrate positioning and catalysis (Fig.4B). Other residues within the binding pocket established hydrophobic interactions with the alkyl hydrophobic chain of BAEE, indicating that hydrogen bonding and hydrophobic interactions are essential for complex formation (Fig.4B).

In the Ac-trypsin-BAEE complex, BAEE occupied a similar binding position and was surrounded by comparable residues, including His40, Leu81, Asp84, Asp171, Ser172, Cys173, Gln174, Gly175, Ser177, Ser192, Trp193, and Gly194 (Fig.4C). Additional nonpolar interactions involving Cys173, Gly196, and Gly204 were observed in Ac-trypsin, suggesting a shift toward hydrophobic mediation of binding (Fig.4C). Notably, BAEE formed three hydrogen bonds in native trypsin, with His40 (3.16 Å), Ser172 (3.09 Å), and Ser177 (2.74 Å) (Fig.4D). In contrast, only two hydrogen bonds were formed in Ac-trypsin, with Asp171(3.02 Å) and Gly196 (3.00 Å) (Fig.4D). The loss of direct hydrogen bonding with catalytic residues His40 and Ser177 indicates that acetylation disrupts the optimal interactions network required for efficient catalysis. This reduction in both the number and strategic placement of hydrogen bonds likely contributes to the decreased enzymatic activity.

### 3.5 MD unveil an allosterically rewired state in the acetylated trypsin-substrate complex

**Fig. 5:**
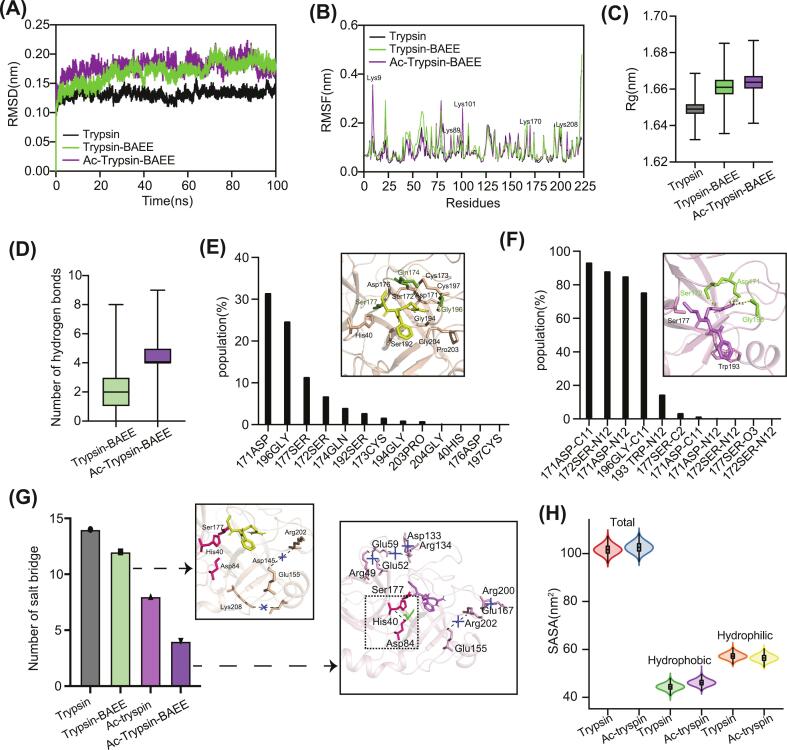
**Molecular dynamics analysis reveals the allosteric mechanism of acetylation-induced functional impairment in trypsin. (A)** RMSD trajectories of trypsin (black), trypsin-BAEE complex (green), and Ac-trypsin-BAEE complex (purple) indicate increased global flexibility in the acetylated complex. **(B)** RMSF profiles identify acetylated lysine residues as key sites of enhanced flexibility, indicating localized structural perturbation. **(C)** Rg values show structural expansion in both trypsin-BAEE and Ac-trypsin-BAEE complexes compared to the trypsin alone. (**D)** Total number of hydrogen bonds between BAEE and trypsin increases in the acetylated complex. **(E, F)** Number of specific hydrogen bonds between the substrate and S1 pocket residues of trypsin (E) and Ac-trypsin (F), indicating reduced yet more stable interactions upon acetylation. **(G)** Salt bridge analysis reveals disruption of the native electrostatic network and formation a new interaction between His40 and Asp84, distorting the catalytic trad geometry. **(H)** SASA analysis conforms a more expanded and solvated conformation in the Ac-trypsin-BAEE complex. (For interpretation of the references to colour in this figure legend, the reader is referred to the web version of this article.)

To elucidate the atomistic mechanisms underlying the acetylation-induced functional impairment, we conducted comprehensive MD simulations of native and acetylated trypsin in complex with the substrate analog BAEE. Global stability analysis revealed that acetylation induces a pronounced shift in the enzyme's conformational energy landscape. The acetylated trypsin-BAEE complex exhibited slightly higher backbone RMSD and Rg compared to the native complex (Fig. 5A, B), indicating global expansion and reduced structural compactness. This expansion was further corroborated by a concomitant increase in SASA (Fig. 5H), confirming a more solvated and relaxed conformational state even in the substrate-bound form. RMSF analysis showed that while the overall fluctuation profiles were conserved, a marked increase in flexibility occurred specifically at the acetylated lysine residues (Fig. 5B), identifying these modification sites as the precise origins of the structural perturbation that propagates through the protein matrix.

Notably, detailed analysis of protein-substrate interactions revealed a decoupling between non-specific and those critical for catalysis. The total number of hydrogen bonds between BAEE and acetylated trypsin increased (Fig. 5D), likely due to enhanced solvent bridging within the expanded binding interface. However, the number of direct, specific hydrogen bonds between the substrate's guanidinium group and key residues lining the S1 pocket was substantially reduced in the acetylated complex (Fig. 5F). Intriguingly, the remaining hydrogen bonds exhibited higher population and stability, suggesting the formation of a new, low-productive energy well that stabilizes a suboptimal enzyme state. Most critically, the native salt-bridge network within the active site was disrupted upon acetylation. In its place, a new interaction between the catalytic residues His40 and Asp84 emerged and stabilized (Fig. 5G). This specific structural alteration distorts the optimal geometry of the catalytic triad, providing an atomic-resolution explanation for the impaired proton transfer efficiency and the experimentally observe reduction in enzymatic activity.

**Fig. 6:**
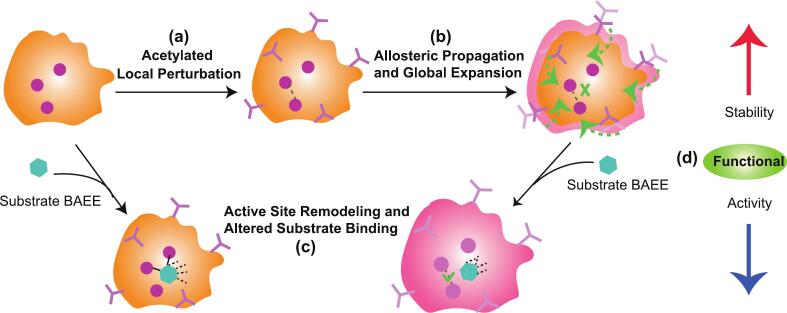
**Proposed mechanistic model of acetylation-induced functional modulation in trypsin.** (a) Local structural perturbation initiated by lysine acetylation. (b) Allosteric propagation of structural changes leading to global expansion of the trypsin structure. (c) Remodeling of the active site geometry and alteration of substrate binding characteristics. (d) Final functional outcome showing reduced catalytic activity despite enhanced structural stability.

In summary, our MD simulations delineate a clear allosteric pathway (Fig. 6): lysine acetylation introduces local flexibility, triggering a global expansion of the trypsin structure. This expansion stabilizes a suboptimal enzyme-substrate complex characterized by abundant, yet non-specific, solvent-mediated hydrogen bonds. The perturbation culminates in the active site through precise restructuring of the catalytic residues, notably the formation of a non-native His40-Asp84 interaction, which misaligns the catalytic apparatus and abrogates function, thereby establishing a classic mechanism of allosteric effect.

## 4 Conclusions

Our work provides a comprehensive mechanistic understanding of how lysine acetylation modulates the structure, stability, and function of trypsin. Our results show that acetylation preserves approximately 80% activity while enhancing stability. In contrast, PEGylation and polymer conjugation often severely impair catalytic efficiency due to steric hindrance (Zuma et al., 2022), and immobilization introduce mass-transfer limitations and altered kinetics(Goharrizi et al., 2025). Unlike time-intensive mutagenesis approaches(Romero & Arnold, 2009), acetylation is a simple one-step chemical modification that targets multiple lysines without disrupting the protein fold. The enhanced structural stability is attributed to reduced autolytic cleavage susceptibility, particularly at key arginine and lysine residues. Conversely, catalytic impairment arises from allosteric perturbations that disrupt the precise geometry of the active site. Notably, MD simulations revealed that acetylation induces global conformational expansion and alters dynamic fluctuations, leading to the formation of a catalytically suboptimal substrate-binding mode characterized by increased non-specific hydrogen bonding and loss of specific interactions essential for transition-state stabilization. Crucially, the distortion of the catalytic triad, specifically the aberrant interaction between His40 and Asp84, confirms an allosteric mechanism through which peripheral chemical modifications propagate to functional residues. These findings highlight a trade-off between stability and activity post-acetylation, with acetylated trypsin exhibiting markedly extended functional longevity alongside moderate reduction in enzymatic activity. The insights gained herein not only elucidate the structural and dynamical basis of trypsin regulation by post-translational modification but also support the rational design of enzyme variants with tailored stability and activity profiles for industrial applications in food processing, detergents, and biotechnology.

## CRediT authorship contribution statement

**Xiaozhan Qu:** Writing – review & editing, Writing – original draft, Visualization, Validation, Supervision, Software, Methodology, Investigation, Funding acquisition, Data curation. **Tengfei Liu:** Writing – review & editing, Validation. **Yalong Xu:** Software, Conceptualization. **Chen Wang:** Validation, Formal analysis, Data curation. **Xueao Zheng:** Validation, Data curation. **Yixiao Zhang:** Validation, Data curation. **Peijian Cao:** Project administration. **Qiansi Chen:** Writing – review & editing, Supervision, Project administration, Funding acquisition, Conceptualization.

## Declaration of competing interest

The authors declare that they have no known competing financial interests or personal relationships that could have appeared to influence the work reported in this paper.

## Data Availability

No data was used for the research described in the article.

## Appendix A Supplementary data

Supplementary material: Fig. S1 Purification and characterization of recombinant trypsin. (A) SDS-PAGE analysis of recombinant trypsin expression: lanes 1, soluble fraction after cell disruption; lane 2, insoluble fraction (inclusion body). (B) Schematic workflow of trypsin purification from inclusion body refolding to final purification. (C) Elution profile from the first Ni-NTA affinity chromatography step. (D) Elution profile from the second Ni-NTA affinity chromatography step. (E) A size-exclusion chromatography profile of purified trypsin. Fig. S2 Sequence and structural features of trypsin. (A) Amino acid sequence and secondary structure features of trypsin. Secondary structure elements (β-strands, α-helices) are annotated above the sequence. (B) Structural representation of trypsin with lysine and arginine residues shown as red and blue sticks, respectively. (C) Electrostatic surface potential comparison between native trypsin and Ac-trypsin calculated using APBS, demonstrating similar overall charge distribution despite lysine acetylation. Fig. S3 Analysis of residue interactions in native and acetylated trypsin. (A) Structural representation of trypsin shown as surface with lysine and neighboring residues exhibiting high RMSF values shown in green sticks. (B) Comparison of specific hydrogen bond populations between native and acetylated trypsin, highlighting key interactions involving catalytic and structural residues. (C, D) Contact map analysis showing mean smallest inter-residue distances, revealing altered residue proximity patterns in AC-trypsin (D) compared to the native form (C). Fig. S4 Biochemical characterization of trypsin and its acetylated form. (A) CD spectra of native trypsin (black), trypsin with BAEE (green), and Ac-trypsin with BAEE (purple). The near superimposition of the three spectra indicates that substrate binding does not induce significant changes in the overall secondary structure of native and acetylated trypsin. (B, C) BAEE-based activity comparison of recombinant trypsin, commercial trypsin, and acetylated commercial trypsin. Recombinant trypsin exhibited the highest specific activity (423,000 U/mL), whereas the activity of commercial trypsin (279,750 U/mL) was significantly reduced after acetylation (26,700 U/mL). This demonstrates that lysine acetylation directly and consistently decreases trypsin activity, independent of enzyme source. Image 1
