# Supplementary material for: Molecular basis of trypsin's autolysis resistance acetylation for sustained enzymatic activity
Source: Food Chem X. 2026 Feb 6;34:103619. doi: 10.1016/j.fochx.2026.103619 (PMC12925162; doi:10.1016/j.fochx.2026.103619)
Supplement: Supplementary file 1 — Supplementary material: Fig. S1 Purification and characterization of recombinant trypsin. (A) SDS-PAGE analysis of recombinant trypsin expression: lanes 1, soluble fraction after cell disruption; lane 2, insoluble fraction (inclusion body). (B) Schematic workflow of trypsin purification from inclusion body refolding to final purification. (C) Elution profile from the first Ni-NTA affinity chromatography step. (D) Elution profile from the second Ni-NTA affinity chromatography step. (E) A size-exclusion chromatography profile of purified trypsin. Fig. S2 Sequence and structural features of trypsin. (A) Amino acid sequence and secondary structure features of trypsin. Secondary structure elements (β-strands, α-helices) are annotated above the sequence. (B) Structural representation of trypsin with lysine and arginine residues shown as red and blue sticks, respectively. (C) Electrostatic surface potential comparison between native trypsin and Ac-trypsin calculated using APBS, demonstrating similar overall charge distribution despite lysine acetylation. Fig. S3 Analysis of residue interactions in native and acetylated trypsin. (A) Structural representation of trypsin shown as surface with lysine and neighboring residues exhibiting high RMSF values shown in green sticks. (B) Comparison of specific hydrogen bond populations between native and acetylated trypsin, highlighting key interactions involving catalytic and structural residues. (C, D) Contact map analysis showing mean smallest inter-residue distances, revealing altered residue proximity patterns in AC-trypsin (D) compared to the native form (C). Fig. S4 Biochemical characterization of trypsin and its acetylated form. (A) CD spectra of native trypsin (black), trypsin with BAEE (green), and Ac-trypsin with BAEE (purple). The near superimposition of the three spectra indicates that substrate binding does not induce significant changes in the overall secondary structure of native and acetylated trypsin. (B, C) BAEE-base [file mmc1.docx]

****Supplementary** Data**

**
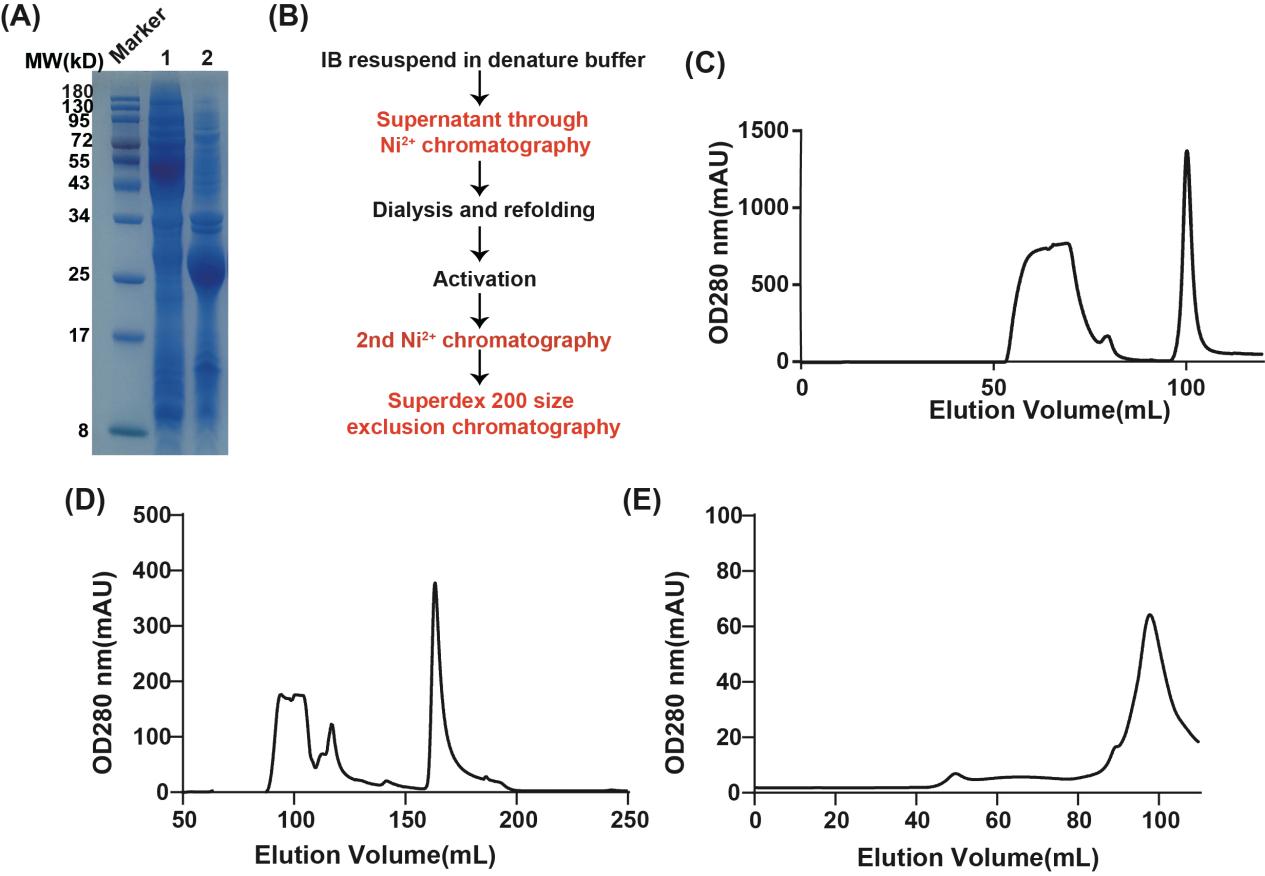
**

**Fig.S1 Purification and characterization of recombinant trypsin. (A)** SDS-PAGE analysis of recombinant trypsin expression: lanes 1, soluble fraction after cell disruption; lane 2, insoluble fraction (inclusion body). **(B)** Schematic workflow of trypsin purification from inclusion body refolding to final purification. **(C)** Elution profile from the first Ni-NTA affinity chromatography step. **(D)** Elution profile from the second Ni-NTA affinity chromatography step. **(E)** A size-exclusion chromatography profile of purified trypsin.

**
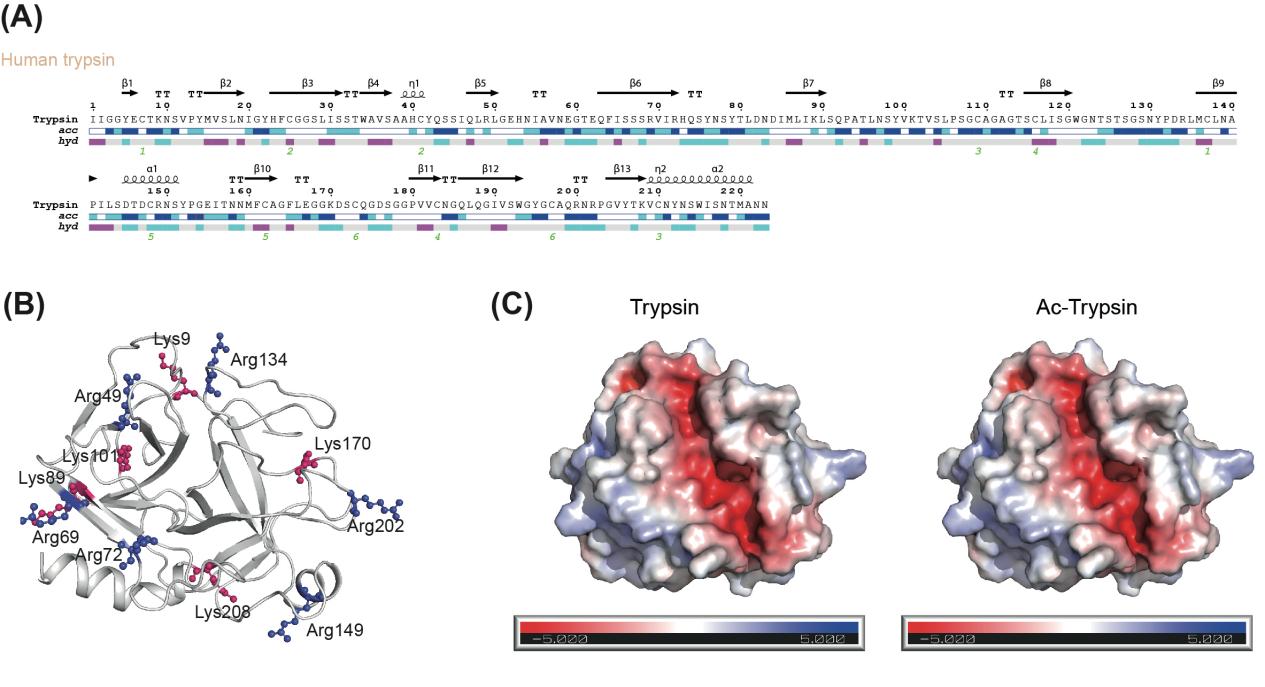
**

**Fig. S2 Sequence and structural features of trypsin. (A)** Amino acid sequence and secondary structure features of trypsin. Secondary structure elements (β-strands, α-helices) are annotated above the sequence. **(B)** Structural representation of trypsin with lysine and arginine residues shown as red and blue sticks, respectively. **(C)** Electrostatic surface potential comparison between native trypsin and Ac-trypsin calculated using APBS, demonstrating similar overall charge distribution despite lysine acetylation.


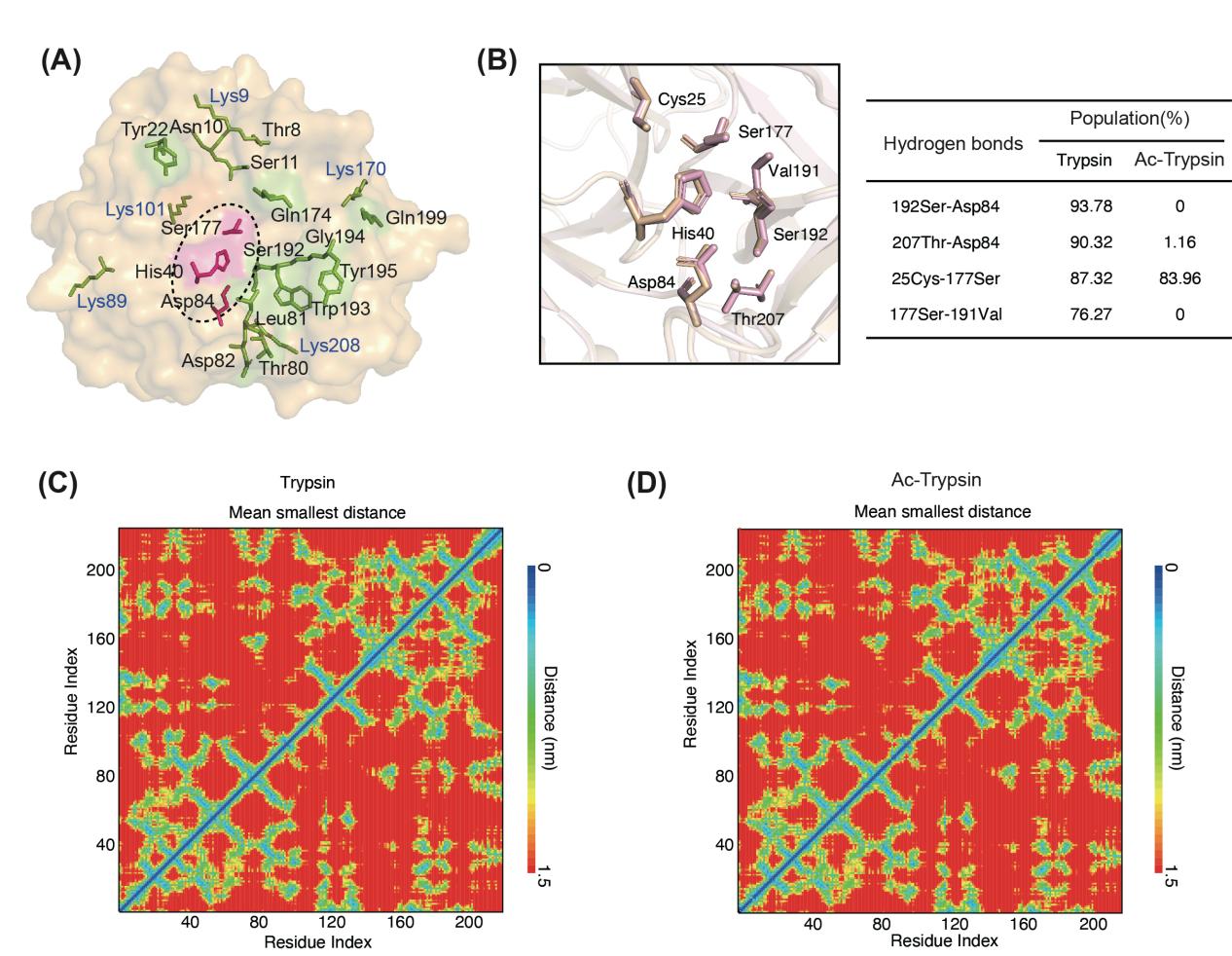


**Fig.S3 Analysis of residue interactions in native and acetylated trypsin. (A)** Structural representation of trypsin shown as surface with lysine and neighboring residues exhibiting high RMSF values shown in green sticks. **(B)** Comparison of specific hydrogen bond populations between native and acetylated trypsin, highlighting key interactions involving catalytic and structural residues. **(C, D)** Contact map analysis showing mean smallest inter-residue distances, revealing altered residue proximity patterns in AC-trypsin (D) compared to the native form (C).

**
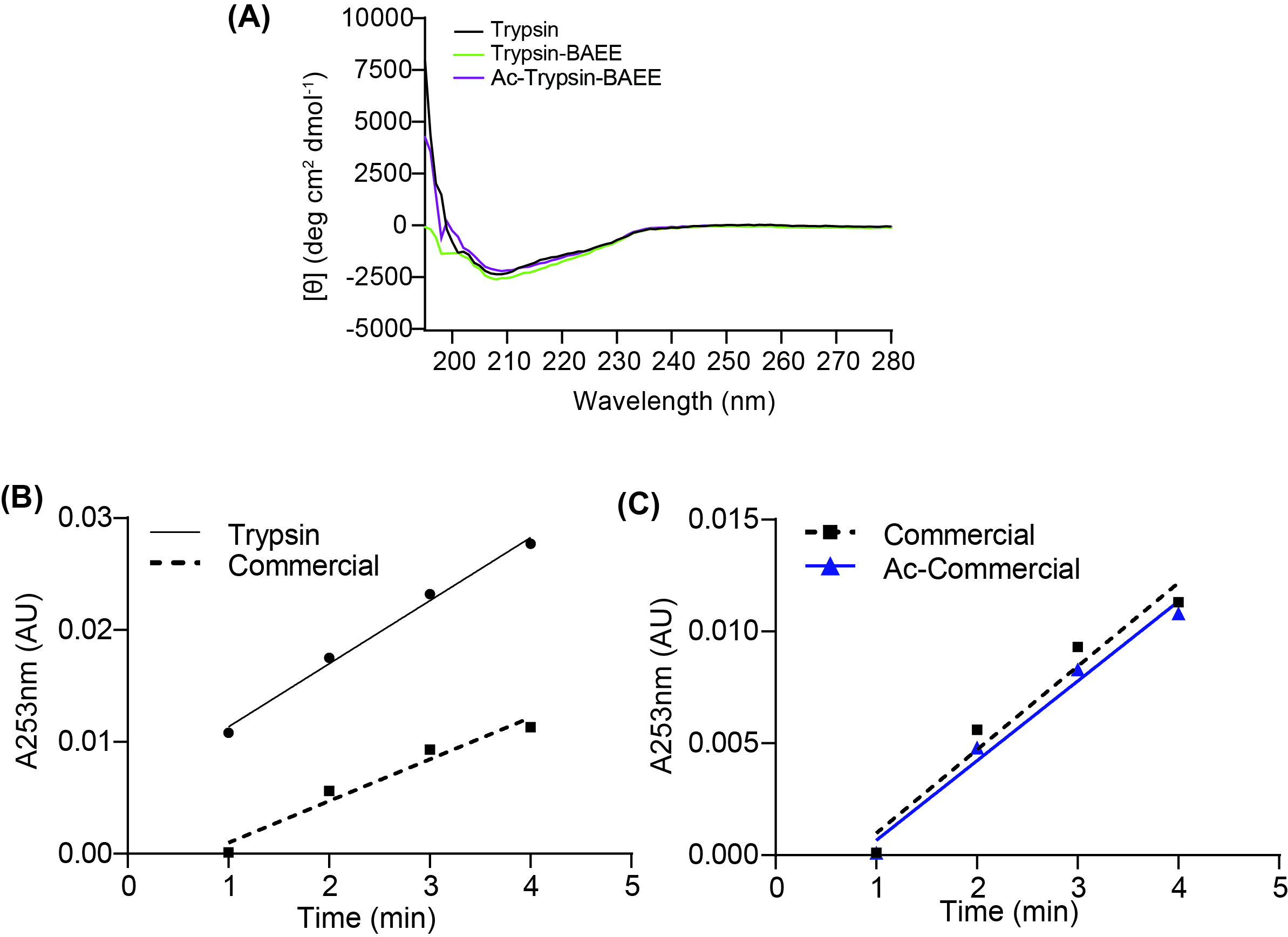
Fig.S4 Biochemical characterization of trypsin and its acetylated form. (A)** CD spectra of native trypsin (black), trypsin with BAEE (green), and Ac-trypsin with BAEE (purple). The near superimposition of the three spectra indicates that substrate binding does not induce significant changes in the overall secondary structure of native and acetylated trypsin. **(B, C)** BAEE-based activity comparison of recombinant trypsin, commercial trypsin, and acetylated commercial trypsin. Recombinant trypsin exhibited the highest specific activity (423,000 U/mL), whereas the activity of commercial trypsin (279,750 U/mL) was significantly reduced after acetylation (26,700 U/mL). This demonstrates that lysine acetylation directly and consistently decreases trypsin activity, independent of enzyme source.
